# DFT calculation of Ac^3+^ and Bi^3+^ complexation with hybrid chelator 3p-*C*-DEPA for targeted alpha therapy

**DOI:** 10.1038/s41598-026-35633-z

**Published:** 2026-01-29

**Authors:** Danni Ramdhani, Hiroshi Watabe, Stephen Ahenkorah, Rina F. Nuwarda, Ari Hardianto, Regaputra S. Janitra

**Affiliations:** 1 Department of Pharmaceutical Analysis and Medicinal Chemistry, Faculty of Pharmacy, Universitas Padjadjaran, Jl Ir. Soekarno KM 21, Jatinangor, 45363 Indonesia; 2 Group of Theranostic Indonesia (GATE-IN), Universitas Padjadjaran, Jl. Ir. Soekarno KM 21, Jatinangor, 45363 Indonesia; 3 Division of Radiation Protection and Safety Control, Research Center for Accelerator and Radioisotope Science (RARiS), Tohoku University, Sendai, Japan; 4 Department of Radiology, The University of Iowa, Iowa City, IA 52242 USA; 5 Department of Chemistry, Faculty of Mathematics and Natural Sciences, Universitas Padjadjaran, Jl. Raya Bandung - Sumedang km 21, Jatinangor, 45363 West Java Indonesia; 6 Research Center for Molecular Biotechnology and Bioinformatics, Universitas Padjadjaran, Bandung, 40132 Indonesia

**Keywords:** DFT calculation, Stability constant, Reactivity, Alpha-particle, 3p-*C*-DEPA, Hybrid chelator, Computational models, Computational chemistry

## Abstract

The stability constant (logK_1_) and reactivity are ultimately the most crucial components to consider during the evaluation and selection of chelators to match with a specific radiometal ion for usage in radiopharmaceutical applications. These components evaluate the thermodynamic stability of the radiometal-chelator complex. Additionally, the effectiveness of chelator in binding with radiometal ions with relatively large atomic radii (e.g., ^213^Bi^3+^ and ^225^Ac^3+^) coupled with charge-diffuse properties result in weaker metal-ligand interactions, and this poses challenges in chelator development. The (2-[(carboxymethyl)]5-(4-nitrophenyl-1-[4,7,10-tris(carboxymethyl)-1,4,7,10-tPentan-2-yl) amino] acetic acid (3p-*C*-DEPA) is a new hybrid chelator designed for potential radio-complexation applications in radio-theranostics and preclinical data has shown great promise for this chelating ligand. Hence, this study investigates the stability constant and chemical reactivity descriptors of the complex generated between 3p-*C*-DEPA and the α-emitting radioisotopes ^213^Bi^3+^ and ^225^Ac^3+^ as well as the β-emitting particle ^177^Lu^3+^ for the first-time using density functional theory (DFT) calculations. The method employs two functional densities, MO6-HF and B3LYP, using the basis set 6-311G(d)/SDD, alongside the continuous solvation models SMD (solvation model density) and COSMO (conductor-like screening model). The interactions of all radiometals with the hybrid chelator 3p-*C*-DEPA are compared to the benchmark chelator, 1,4,7,10-tetrazacyclodecane-1,4,7,10-tetraacetic acid (DOTA), yielding comprehensive data on the stability constants and based structural features of radiometal-chelator complexes. DFT analysis has shown that the stability of the 3p-*C*-DEPA chelator complex formation is influenced by the atomic radius of the radiometal and the number of nitrogen and oxygen donors, proving to be effective for Ac^3+^ and Bi^3+^, in contrast to Lu^3+^, which shows lower stability constant values.

**Supplementary Information:**

The online version contains supplementary material available at 10.1038/s41598-026-35633-z.

## Introduction

Radioisotopes in the form of radiopharmaceuticals are increasingly used for medical applications in cancer imaging (e.g., gamma and positron emitting particles) and therapy (e.g., alpha, beta, and auger electrons)^1,2^. Bifunctional chelators (BFCs), which are essentially chelators with reactive functional groups that can covalently bind to targeting vectors (such as a peptide or antibody), are ligands commonly utilized in radiometal-based radiopharmaceuticals development^3^. BFCs should possess strong thermodynamic stability and rapid radiolabeling kinetics under moderate circumstances^4,5^.

Currently, DOTA is the most utilized chelator in FDA-approved radiopharmaceuticals for cancer diagnosis (e.g., ^68^Ga-DOTA-TATE) and therapy (e.g., ^177^Lu-DOTA-TATE). The conventional radiolabeling method for DOTA requires higher temperatures, typically 30–60 min at 95 °C, which are unsuitable for heat-sensitive vector molecules^6,7^. The thermodynamic stability of radiometal-ion DOTA complexes is inversely correlated with the atomic radius of the metal ion. It is worth mentioning that complexes with larger metal atom centers possess less stability (e.g., Ac^3+^). Furthermore, the kinetic inertness of radiometal ion-chelator complex formation is a critical element in evaluating the suitability of chelating agents^8^. Several investigations have questioned the kinetic stability of ^225^Ac-DOTA complexes, due to the dissociation of ^225^Ac^3+^ from DOTA in vitro and in vivo^9,10^.

Steric factors and electrostatic interactions are the primary factors responsible for complex radiometal-chelator interactions. The very large ionic radius of the metal ion results in the creation of kinetically unstable complexes, as the constancy of the electrostatic forces is directly related to the charge-to-distance ratio^11^. The radius of ions of the metal ion exhibits an inverse relationship with the thermodynamic stability of DOTA-metal ion complexes. Hence, a chelator that efficiently binds and traps these radiometals is crucial for both therapeutic and diagnostic applications^12^.

Finding appropriate chelating agents continues to be a major obstacle in the development of novel ^225^Ac and ^213^Bi radiopharmaceuticals, in addition to availability. Recent research has indicated that a bifunctional analog of macropa, specifically macropa-NCS conjugated with the antibody trastuzumab, and the prostate specific membrane antigen (PSMA) targeting agent complexed with ^225^Ac provided quantitative radiochemical yield at room temperature, demonstrating a significant advantage over DOTA antibody conjugates, which are unable to complex ^225^Ac under mild conditions^13^.

3p-*C*-DEPA (2-[(carboxymethyl)][5-(4-nitrophenyl-1-[4,7,10-tris(carboxymethyl)−1,4,7,10-tetraazacyclododecan-1-yl]pentan-2-yl)amino]acetic acid) is a hybridized form of the macrocyclic DOTA and the acyclic DTPA. It is hypothesized that the macrocyclic and acyclic binding moieties will rapidly form a stable complex with a radiometal at mild temperature conditions. 3p-*C-*DEPA conjugated to trastuzumab bound to ^205/206^Bi rapidly, and the corresponding [^205/206^Bi] Bi-3p-*C*-DEPA-trastuzumab complex was stable in human serum for four days (d). ^11,12^ The 3p-*C-*DEPA has also been radiolabeled with ^225^Ac however, the stability profile was not provided.

**Fig. 1:**
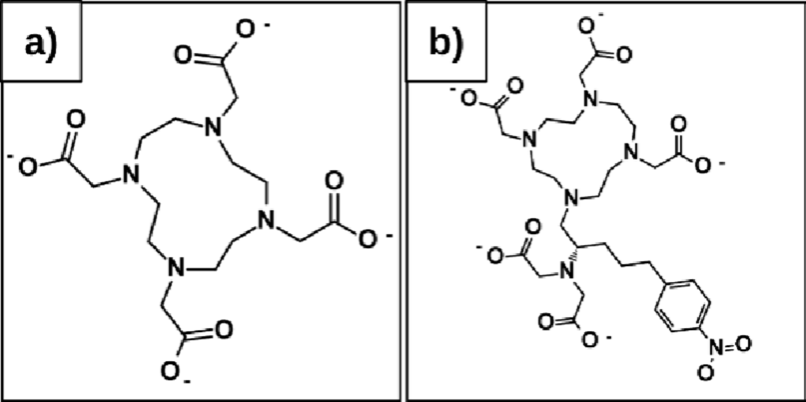
(**a**) DOTA; coordination number = 8; (**b**) 3p-*C*-DEPA; coordination number = 10.

This study presents update insights into the radiolabeling properties of the hybrid chelator 3p-*C*-DEPA, Utilizing DOTA as a reference in DFT computations to assess the stability constants and reactivity of the radiometal-chelator complex. The structure of DOTA and 3p-*C*-DEPA are in Fig. 1. We compared the stability and reactivity features in the formation of chelators with beta-emitting particles ^177^Lu^3+^, and alpha-emitting particles ^213^Bi^3+^ and ^225^Ac^3+^. Ab initio models integrating DFT of formed complexes are utilized to examine Energy persistence and structure-property correlations from a quantum mechanics viewpoint.

DFT simulations were applied to identify the thermodynamic stable parameters of the complexes and to identify the stable structures formed; the vibrational frequencies were examined to confirm the absence of negative frequencies. The implicit solvation model has been developed as a methodology for standard conditions in radiosynthesis and for evaluating the substances according to stability of radio-complexes during in vitro testing^13,14^. We selected M06-HF/6-311G(d) and B3LYP/6-311G(d) as the density functional and basis sets, combining the continuum solvation models SMD and COSMO).

## Computational methods

### Experimental section

#### Computational details

All quantum chemistry computations were conducted with the Gaussian 16 C.01 software (Win64bit) and Gauss View6 (Win 64 bit), and the official URL link of gaussian 16 is https://gaussian.com/gaussian16/^15^. This Gaussian software is officially licensed by Tohoku University. The ChemCraft software was employed for the graphical visualization of the structure, with the URL link https://www.chemcraftprog.com/. The hybrid meta-exchange-correlation full-Hartree–Fock functionals (M06-HF) and B3LYP were selected as the functional. The 6-311G(d) basis set was applied for DOTA and 3p-*C*-DEPA, whereas the SDD basis set were used for the radiometal ions in the formation complexes. Furthermore, implicit solvation simulations, including COSMO and SMD, were utilized to simulate standards for radiolabeling and the integrity of radiometal-complex in vitro. These models assist the consideration of solvation effects on the stability and characteristics of the radiometal-complexes^16^.

#### DFT calculations

This study examined the radiometal-ligand formed between Lu^3+^, Bi^3+^ and Ac^3+^ metals with 3p-*C*-DEPA, using DOTA as a reference. All structure optimizations and frequency computations were carried out in the gaseous state. The frequency computation data was applied to determine the comprehensive Correction for enthalpy and entropy at T = 298.15 K, in combination with verification that the structure of the geometry represents a minimum (absence of imaginary frequencies) on the potential energy surfaces. Calculation of the gas stage’s free energy for all structures and the difference ΔG°g, which will be combined with all the energy from DFT.

This study computed single-point liquid solvation free energy, ΔG*solv, harnessing gas-phase structures and all the COSMO and SMD solvation models. The thermodynamic cycle was finalized for calculating the stability constant, Log K_1_, and the free energy changes of the liquid phase, ΔGaq.

#### Characteristic based on theoretical DFT

The structural features obatained by DFT (chemical hardness, η; and softness, S) were calculated using the subsequent equations:

1 $$\:\:\:\:{\upeta\:}=\frac{(\mathrm{I}\mathrm{P}-\mathrm{E}\mathrm{A})}{2}\:\:\:\mathrm{S}=\frac{1}{2{\upeta\:}}$$

Ionization potential (IP) and electron affinity (EA) were computed using DFT computations based on the frontier orbital energies, specifically the highest occupied molecular orbital (HOMO) and the lowest unoccupied molecular orbital (LUMO)^17,18^.

## Results and discussion

### DFT calculation

#### Thermodynamic cycle for determining stability constants

A stability constant in coordination chemistry denotes the equilibrium value for the formation of a complex in solvation. The complex commonly referred to as a stability constant or formation constant. This work examined the ratio of a 1:1 complex through the interaction of chelators (3p-*C*-DEPA and DOTA) with radiometals. The determination of the stability value K_1_ from the 1:1 metal ion/chelator ratio under stable stat; e is associated with the variation in the Gibbs free energy reactions in the solution, ΔGaq. The kinetic energy of the radiometal-ligand reaction is calculated by specific log K_1_ values, and the differences between the log K_1_ values of two radiometal ions signify the level of selectivity^19,20^.

2 $$\:{{\left[\mathrm{M}{\left({\mathrm{H}}_{2}\mathrm{O}\right)}_{m}\right]}^{x}}_{\left(aq\right)}+{{\mathrm{L}}^{y}}_{\left(aq\right)}\:\rightleftharpoons\:{{\left[\mathrm{M}\mathrm{L}{\left({\mathrm{H}}_{2}\mathrm{O}\right)}_{m-n}\right]}^{x+y}}_{\left(aq\right)}\:+\:\mathrm{n}{{\mathrm{H}}_{2}\mathrm{O}}_{\left(l\right)}\:\:\:\:log{\mathrm{K}}_{1}=\mathrm{l}\mathrm{o}\mathrm{g}\:\frac{\left[{\mathrm{M}\mathrm{L}{\left({\mathrm{H}}_{2}\mathrm{O}\right)}_{m-n}}^{x+y}\right]}{\left[{\mathrm{M}{\left({\mathrm{H}}_{2}\mathrm{O}\right)}_{m}}^{x}\right]\left[{L}^{y}\right]}=\:\frac{-{\Delta\:}{\mathrm{G}}_{\:\mathrm{a}\mathrm{q}}}{2.303\:\mathrm{R}\mathrm{T}}$$

**Fig. 2:**
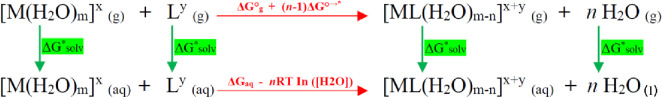
Thermodynamic cycle used to calculate ΔGaq.

The calculation of ΔGaq is determined by the thermodynamic cycle illustrated in Fig. 2.

The free-energy corrections of the radiometal and chelator interactions in the gas phase are denoted by the symbol ΔG°g in this process, ΔG*solv represents the free energy required for solving 1 mol of compound from a gas phase into a liquid state^21^. The model below determines the amount of ΔG°g for normal ideal gas settings at 1 atm (24.46 mol/L) to 1 M (1 mol/L).

$$\Delta {\mathrm{G}}^{{\mathrm{o}}\rightarrow*} = {-}{\mathrm{T}}\Delta {\mathrm{S}}^{{0}\rightarrow*}= {\text{ RT In }}\left( {{\mathrm{Vo}}/{\mathrm{V}}*} \right) = {\mathrm{R}}.{\mathrm{T}}.{\mathrm{In}}\left( {{\mathrm{24}}.{\mathrm{46}}} \right) = {\text{ 1}}.{\text{89 kcal}}/{\text{mol }}\left( {{\text{T }} = {\text{ 298}}.{\text{15 K}}} \right)$$

Correction of calculations is particularly crucial when pure solvent H_2_O_(l)_ is designated as the reference state for the solvent, with the system’s state expressed as G_aq_* = G_aq_* + RT ln ([H_2_O]). The free energy shift needed for converting the solvent from a standard-state solution-phase concentration of 1 M to a standard-state pure liquid concentration of 55.34 M is determined by the equation RT ln([H_2_O]) = 2.38 kcal/mol^19,22^.

We completed DFT computations to determine the stability constants of complexes created by combining metals (Lu^3+^, Bi^3+^ and Ac^3+^) with 3p-*C*-DEPA chelator, respectively. Furthermore, the stability constants of the DOTA complex for each radiometal ion were computed and applied as a reference point. To determine the stability constants of complexes formed by the metals Lu^3+^, Bi^3+^ and Ac^3+^ with 3p-*C*-DEPA, we performed DFT calculations using DOTA as a reference. Chelators will form complexes with metals exhibiting oxidation states of + 3 and a coordination number (CN) of 9.

The DFT analysis of the Ac^3+^ ion with 4–11 water molecules showed that [Ac (H_2_O)_9_]^3+^is the most stable in both the gas phase and aqueous phase (COSMO model), which provides the basis for this choice of CN 9^23^. Similarly, the selection of CN for Lu^3+^ was based on a geometric stability analysis of the water exchange process for Lu^3+^ ions. Also, several studies have reported that Lu^3+^ exhibit a CN 9 based on the stability of its geometry and crystal structure. ^22,24^

Targeted alpha therapy (TAT), using ^213^Bi (T_1/2_ = 45.6 min), can be readily obtained from ^225^Ac/^213^Bi generators and it emits a single α-particle during its decay process making it an interesting radioisotope for TAT. Bi^3+^ exhibits variable coordination number (3–10) and this is based on the characteristics of the donor atoms, polydentate ligand, and the solvent. Moreover, in highly acidic environments, Bi^3+^ undergoes hydrolysis at a relatively fast rate in aqueous solution. Therefore, it is challenging to study Bi^3+^ complexes in aqueous solutions due to the formation of hydrolysis products. Several research has suggested a 9-coordinate configuration form of Bi^3+^with an ionic radius ranging from 1.00 to 1.20 Å^25,26^.

This study utilized MO6-HF as a hybrid density functional in the DFT calculation, gaining an advantage on its strengths for assessing the main classes thermochemistry, thermochemical kinetics, noncovalent interactions, excited states, and transition metals^27^. This study also used B3LYP as a comparison of hybrid density functionals that have been commonly used. Furthermore, MO6-HF demonstrates a remarkable self-interaction error, as evidenced by its Low averaged means uncorrected errors (in kcal/mol), especially in comparison to the commonly employed functionals PBE and B3LYP^28^.

**Fig. 3:**
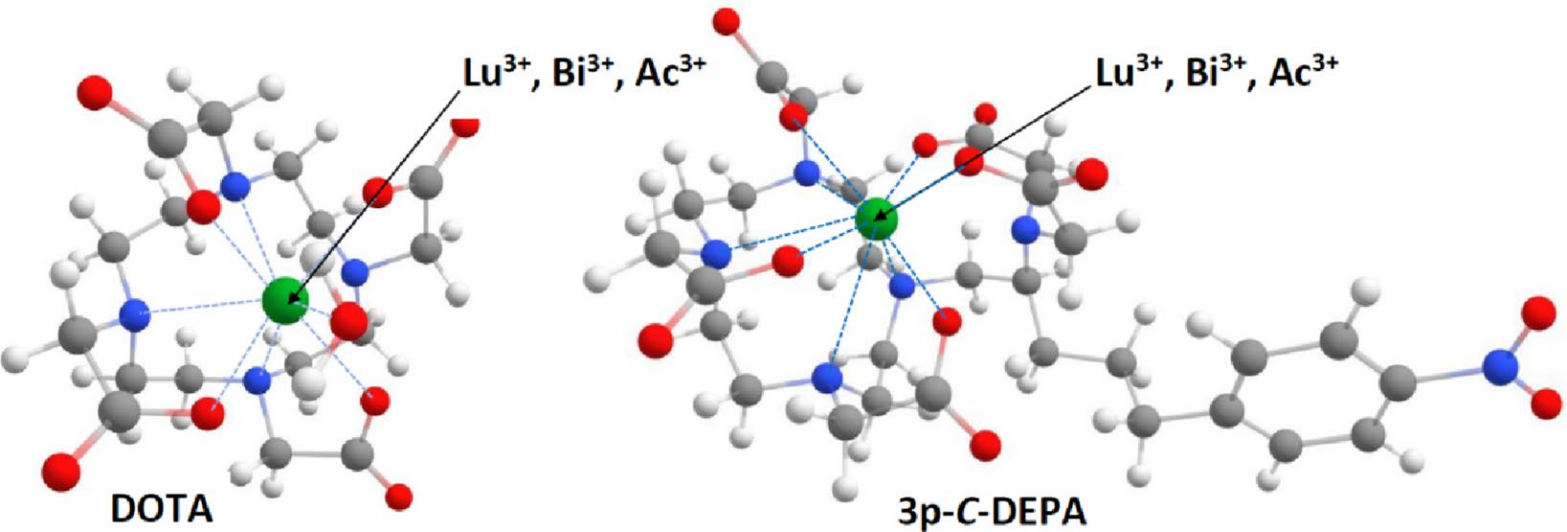
The structure of the 3p-*C*-DEPA-radiometal ion complexes in comparison with DOTA complexes. The pictures demonstrate intermolecular lengths between nearby heteroatoms and radiometal ions, indicated by blue lines, alongside the geometrical form of 3p.-*C*-DEPA and DOTA resulting from geometry optimization.

The geometry optimization and frequency calculation of the complex structure yield information regarding the atomic positions, bond connectivity with radiometal, and functional groups that affect the stability and reactivity of the generated complex (Fig. 3).

We computed single-point liquid solvation free energies, ΔG*solv, utilizing gaseous phase structures, and employed the two models SMD model and COSMO to assess electrical interactions between the molecules and a solvent. The estimations of stability values might be improved through the application of highly computational methods, such as explicitly solvents quantum computations^29^. Table 1 presents DFT computations for the stability constants (log K_1_) of the radiometal-ligand complex in the gas phase and the solvation models (SMD and COSMO).

In addition, DFT computations obtained the stability values of the metals-DOTA complex, revealing that Ac^3+^ presents the smallest radiosynthesis result in comparison to the radio-complexes formed with Lu^3+^ and Bi^3+^. DOTA (N_4_O_4_) provides Octa-dentate coordinating via quartet tertiary amine nitrogen donors and quartet unique carboxylic acid arms, creating inadequate cavities for capturing Ac^3+^ ions, resulting in a kinetic unstable complexes^10^. Several studies demonstrating the degradation of the kinetics and stability of the ^225^Ac-DOTA complex, both in vitro and in vivo, have generated concerns^8^. Moreover, the DFT calculation results indicate that Ac^3+^ forms the most stable compound with 3p-*C*-DEPA, followed by Bi^3+^. The findings suggested that the α-emitting particles Ac^3+^ and Bi^3+^ exhibit effective binding in the presence of sufficient cavity space, with the number of donor groups being a critical factor. Furthermore, the large ionic radii of Ac^3+^ and Bi^3+^ compared to Lu^3+^ are suspected to reduce repulsion between carboxylic group arms when coordinating with 3p-*C*-DEPAThis aligns with the radiochemical conversion (RCC) described by Ahenkorah et al.. in their radiosynthesis investigations with Ac^3+^. The synthesis of the ^225^Ac^3+^ complex with 3p-*C*-DEPA at a concentration of 1.5 nmol at 25 °C achieved an RCC exceeding 90% while increasing the temperature to 40 °C yielded RCC > 95%.^30^ Increasing concentration and temperature of the chelator greatly enhanced the RCC. These findings showed that the cavity and Deca-dentate coordination via five tertiary amine nitrogen donors and five distinct carboxylic acid groups in 3p-*C*-DEPA are highly effective in trapping Bi^3+^ and Ac^3+^, which possess a larger atomic radius, in contrast to Lu^3+^ which has a smaller atomic radius^11,24^. Song, et al.. has also reported radiolabeling of ^177^Lu^3+^ with 3p-*C*-DEPA, which showed RCC of 87% within 30 minutes. This slow reactions kinetics could be that the Deca-dentate 3p-*C*-DEPA features an excess of donor groups, potentially hindering the rapid formation of a stable Lu^3+^ complex due to repulsive interactions between the carboxylic group arms and potentially result in the [Lu(3p-*C*-DEPA)]^2−^ complex being formed in a reversible process^14^.

**Table 1:** Calculated stability constants (log K_1_) for the metal ion-chelator complexes.

Chelators	Metals	M06-HF/6-311G(d)			B3LYP/6-311G(d)		
		Log K_1_ (Gas)	Log K_1_ (SMD)	Log K_1_ (COSMO)	Log K_1_ (Gas)	Log K_1_ (SMD)	Log K_1_ (COSMO)
[DOTA]^4–^	Lu ^3+^	830.72	41.38	46.13	607.82	45.44	56.04
	Bi ^3+^	833.52	46.95	54.86	611.44	52.55	69.54
	Ac ^3+^	815.83	22.75	28.17	-	-	-
[3p-*C*-DEPA]^5–^	Lu ^3+^	636.69	20.93	29.44	485.44	24.72	29.79
	Bi ^3+^	650.91	40.51	47.66	912.486	45.01	58.81
	Ac ^3+^	637.07	60.76	68.57	-	-	-

We performed comparisons in Tables 2 and 3, and 4between the calculated stability constants (Log K₁) and the experimental Log K₁ and pM values, both of which are commonly used as indicators of the affinity of metal ions for ligands. Log K₁ is directly proportional to pM; higher values indicate stronger metal-ligand affinity^32^. For metal ion complexes with DOTA, we compared the calculated Log K₁ values with experimental Log K₁ and pM data. However, due to limited experimental data for metal ion complexes with 3p-*C*-NETA and 3p-*C*-DEPA, we relied on Log K₁ values estimated from radiochemical conversion (RCC), which technically reflect affinities for specific isotopes only. Additionally, for 3p-*C*-DEPA, we compared the results with experimental labeling data of 3p-*C*-DEPA-trastuzumab^33,34^. Overall, our calculated Log K₁ values are in good agreement with the experimental Log K₁ and pM data.

**Table 2:** Comparison of calculated log K₁ values with experimental log K₁ and pM data for metal ion-DOTA complexes.

Chelator	Metals	M06-HF/SDD/6-311G(d)				B3LYP/SDD/6-311G(d)				Experiment		
		Log K_1_ (Gas)	Log K_1_ (SMD)	Log K_1_ (COSMO)	Ref	Log K_1_ (Gas)	Log K_1_ (SMD)	Log K_1_ (COSMO)	Ref	Log K_1_	pM	Ref
[DOTA]^4−^	Ga^3+^	891.5	46.86	68.18	[34]	666.7	48.0	64.13	^31^	26.1	15.7	^32^
	Tb^3+^	828.3	37.59	49.66	[34]	604.1	39.6	56.73	^31^	24.2	-	^32^
	Lu^3+^	830.7	41.38	46.13	This work	607.8	45.4	56.04	[This Work]	25.4	20.8	^32^
	Bi^3+^	833.5	46.95	54.86	[34]	611.4	52.5	69.54	^31^	30.3	27.0	^32^

*Data based on Ref [32].

**Table 3:** Comparison of calculated log K₁ values with experimental log K₁ data for metal ion–3p-*C*-NETA complexes.

Chelator	Metals	M06-HF/SDD/6-311G(d)				B3LYP/SDD/6-311G(d)				Experiment	
		Log K_1_ (Gas)	Log K_1_ (SMD)	Log K_1_ (COSMO)	Ref	Log K_1_ (Gas)	Log K_1_ (SMD)	Log K_1_ (COSMO)	Ref	Log K_1_^*^	Ref
[3p-*C*-NETA]^4−^	Ga^3+^	644.51	54.56	67.98	^31^	635.23	45.61	60.07	^31^	5.34	^29^
	Tb^3+^	566.10	32.18	39.88	^31^	557.99	29.29	37.91	^31^	5.35	^29^
	Bi^3+^	573.15	42.85	47.66	^31^	568.88	42.89	57.10	^31^	6.28	^29^
	Ac^3+^	556.17	22.55	27.71	^31^	-	-	-	^31^	5.82	^29^

*Calculated based on RCC labeling of radioisotopes at 25 °C. The isotopes involved are Ga-68; Tb-161; Bi-213; and Ac-225.

**Table 4:** Comparison of calculated log K₁ values with experimental log K₁ data for metal Ion–3p-C-DEPA Complexes.

Chelator	Metals	M06-HF/SDD/6-311G(d)				B3LYP/SDD/6-311G(d)				Experiment	
		Log K_1_ (Gas)	Log K_1_ (SMD)	Log K_1_ (COSMO)	Ref	Log K_1_ (Gas)	Log K_1_ (SMD)	Log K_1_ (COSMO)	Ref	Log K_1_^*^	Ref
[3p-*C*-DEPA]^5−^	Bi^3+^	650.91	40.51	47.66	This work	912.49	45.01	58.81	This work	7.11	^33^

*Calculated based on RCC labeling of radioisotopes with 3p-*C*-DEPA-trastuzumab at room temperature.

### Characteristic based on theoretical DFT

Several chemical reactivity indicators have been proposed through research in various aspects of pharmacological sciences, particularly in the context of drugs development. DFT calculations can evaluate the significance of reactive characteristics such as chemistry potential, electronegativity, chemical hardness, softness, and index of electrophilicity as a fundamental component^34^. Ionizing potential signifies the ability of an atom or molecule to relinquish electrons, while electron affinity indicates its capability at attracting electrons. Chemical hardness, associated with the stability of a chemical system, indicates the opposition to modifications in circulation of electrons. The softness, associated with the reactive of the chemical system, is the antithesis of chemical hardness^14,35^. Table 5 presents the reactivity index data, encompassing ionization potential (IP), electron affinity (EA), electro-donating power (ω^−^), electro-accepting power (ω^+^), and net electrophilicity (Δω^±^) for radiometal ions, ligands, and complexes.

**Table 5:** Ionization potential (IP), electron affinity (EA), electron donating capacity (ω^−^), electron accepting capacity (ω^+^), and net electrophilicity (Δω^±^) for radiometal ions (Lu^3+^, Bi^3+^, and Ac^3+^); the ligands (DOTA and 3p-*C*-DEPA); and the complexes were computed using the M06-HF method with the SDD basis set for the radiometal ion and the 6-311G(d) basis set for the other atom.

System	Energy (eV)					
	IP	EA	η	ω^-^	ω^+^	Δω^±^
Lu^3+^	28.485	1.108	13.689	17.106	2.310	2.251
	51.910	22.324	14.793	66.973	29.856	29.841
Bi^3+^	23.197	14.065	4.566	47.898	29.267	29.246
	42.693	33.544	4.575	178.439	140.321	140.315
Ac^3+^	24.060	0.497	11.782	14.010	1.731	1.660
	48.496	18.722	14.887	56.603	22.994	22.977
DOTA^4-^	8.500	−3.086	5.793	2.710	0.003	−0.366
	−1.654	−12.343	5.345	1.751	8.749	8.178
[3p-*C*-DEPA]^5-^	8.053	0.107	3.973	4.632	0.552	0.336
	−2.808	−7.767	2.479	3.304	8.592	8.290
Lu(DOTA)(H_2_O)]^-^	10.433	−1.918	6.176	4.368	0.111	−0.118
	7.976	−4.063	6.020	2.048	0.092	−0.396
[Lu(3p-*C*-DEPA)]^2-^	9.450	0.093	4.678	5.404	0.632	0.447
	4.713	−3.677	4.195	0.815	0.297	−0.929
[Bi(DOTA)(H_2_O)]^-^	7.012	−2.551	4.781	2.233	0.003	−0.445
	4.240	−4.177	4.209	0.542	0.510	−1.334
[Bi(3p-*C*-DEPA)]^2-^	7.181	0.097	3.542	4.131	0.493	0.250
	2.104	−3.531	2.818	0.086	0.799	−10.852
[Ac(DOTA)(H_2_O)]^-^	10.181	0.080	5.051	5.802	0.672	0.499
	8.038	−1.011	4.524	3.686	0.173	−0.098
[Ac(3p-*C*-DEPA)]^2-^	9.640	0.091	4.774	5.509	0.643	0.462
	5.016	−2.730	3.873	1.224	0.081	−0.735

Electronic factors are considered to significantly impact the stability of the [Bi(3p-C-DEPA)]^2−^ complex. Bi^3+^ has a strong tendency to accept electrons and shows a similarity in hardness to 3p-*C*-DEPA. Bi^3+^ and 3p-*C*-DEPA form a soft Lewis acid-base pair, and thus the interaction formed is predicted to have covalent characteristics.

In contrast, for the complexes of 3p-*C*-DEPA with Ac^3+^ and Lu^3+^, electronic factors are less significant for the stability of the complexes. Both are classified as hard Lewis acids and have a low tendency to accept electrons; thus, the driving force for the formation of complexes with 3p-*C*-DEPA is electrostatic interaction^8,36^. The reactivity index calculations for 3p-*C*-NETA show similarities to 3p-*C*-DEPA, but the stability constant of the [Ac(3p-*C*-NETA)(H_2_O)]^−^ complex being lower than that of [Ac(3p-*C*-DEPA)]^2−^ indicates the importance of the role of anionic oxygen donor atoms in the coordination of Ac^3+^ with the ligand. This strengthens the hypothesis that the issue of kinetic lability in Ac^3+^ complexes, caused by its large ionic radius, can be addressed by raising the amount of anionic oxygen donor atoms. In the case of Lu^3+^, the addition of donor atoms must be taken into account the electrostatic repulsion effect between anionic oxygen atoms during coordination. The higher stability constant of [Lu(DOTA)(H_2_O)]^−^ compared to [Lu(3p-C-DEPA)]^2−^ indicates complex destabilization due to repulsion between donor atoms.

## Conclusion

DFT calculations using M06-HF and B3LYP as density functionals with 6-311G(d)/SDD basis sets were conducted to examine the reactions involved in the complexation of 3p-*C*-DEPA with metal ions Ac^3+^, Bi^3+^, and Lu^3+^. We utilize the implicit solving models SMD and COSMO for modeling the electrostatic interactions between the solute and its solvent, utilizing a thermodynamic cycling method to determine ΔG_aq_, and can significantly eliminate systematic errors in computing protocols.

Our analysis shows that the formation constant of [Ac(3p-C-DEPA)]^2−^ has a more stable value compared to other complexes [Bi(3p-C-DEPA)]^2−^, and [Lu(3p-C-DEPA)]^2−^. In addition, DOTA is used as a reference ligand which appears to be less stable for application with Ac^3+^ radiometal due to its large atomic radius. Generally, ligand-ion and ion-water reactions are determined by the atomic charge and atomic radius of the metal ion, what are the primary elements influencing chelation stability. Ac^3+^ possesses a larger atomic radius, leading to the formation of a kinetically unstable complex. The 3p-*C*-DEPA could be a promising chelator for Ac^3+^ in radiopharmaceutical applications. Hence, further studies are warranted to fully understand 3p-*C*-DEPA for possible future applications in the medical setting.

## Supplementary Information

Below is the link to the electronic supplementary material.

Supplementary Material 1

## Data Availability

The authors declare that the data supporting the findings of this study are available within the paper and its Supplementary Information files. Should any raw data files be needed in another format they are available from the corresponding author upon reasonable request. Source data are provided with this paper. Additional data can be obtained by contacting the corresponding author: d.ramdhani@unpad.ac.id.

## Declarations

### Associated content

The Supporting Information is available free of charge. Calculated energies from the optimized structures of complex compiled ΔG°_g_, ΔG_aq_, and log K_1_ calculated in this work; DFT-optimized structure and cartesian coordinate of [Lu(DOTA)(H_2_O)]^-^, and [Lu(3p-*C*-DEPA)]^2-^ complex; DFT-optimized structure and cartesian coordinate of [Bi(DOTA)(H_2_O)]^-^, and [Bi(3p-*C*-DEPA)]^2-^ complex; DFT-optimized structure and cartesian coordinate of [Ac(DOTA)(H2O)]-, and [Ac(3p-C-DEPA)]2-complex (PDF).
