# Supplementary material for: DFT calculation of Ac3+ and Bi3+ complexation with hybrid chelator 3p-C-DEPA for targeted alpha therapy
Source: Sci Rep. 2026 Jan 29;16:6587. doi: 10.1038/s41598-026-35633-z (PMC12913990; doi:10.1038/s41598-026-35633-z)
Supplement: Supplementary file 1 — Supplementary Material 1 [file 41598_2026_35633_MOESM1_ESM.pdf]

Supplementary Information for:

DFT Calculation of $^{225}Ac^{3+}$, $^{213}Bi^{3+}$ and $^{177}Lu^{3+}$ with Hybrid Chelator 3p-*C*-DEPA to Predict the Stability Constants and Reactivities: Challenges and Perspectives

Danni Ramdhani[1], Hiroshi Watabe[2], Stephen Ahenkorah[3], Rina F. Nuwarda[1], Ari Hardianto[4], Regaputra S. Janitra[5]*

[1]Department of Pharmaceutical Analysis and Medicinal Chemistry, Faculty of Pharmacy, Universitas Padjadjaran, Jl. Raya Bandung-Sumedang km 21, Jatinangor, 45363, West Java, Indonesia

[2]Division of Radiation Protection and Safety Control, Research Center for Accelerator and Radioisotope Science (RARiS), Tohoku University, Sendai, Japan

[3]Carver College of Medicine, Department of Radiology, Theranostic Lab, University of Iowa Hospitals and Clinics, 200 Hawkins Drive, 3916 JPP, Iowa City, lA 52242, United States

[4]Department of Chemistry, Faculty of Mathematics and Natural Sciences, Universitas Padjadjaran, Jl. Raya Bandung - Sumedang km 21, Jatinangor, 45363, West Java, Indonesia

[5]Research Center for Molecular Biotechnology and Bioinformatics, Universitas Padjadjaran, Bandung, 40132, Indonesia

AUTHOR INFORMATION

Corresponding Author

*E-mail: d.ramdhani@unpad.ac.id

Table S1. Calculated energies from the optimized structures of complex

Complex	Functional/ Basis Sets[a]	Electronic Energy (Hartree)	Zero-point Energy (Hartree)	Thermal Free Energy Correction (kcal/mol)	Total Entropy (cal/mol/K)	ΔG*solv (COSMO) (kcal/mol)	ΔG*solv (SMD) (kcal/mol)
$[Ac(H_2O)_9]^{3+}$	M06-HF/ 6-311G(d)	-1050.824	-1050.594	-1050.650	175.375	-1885.643	-1885.693
	B3LYP/ 6-311G(d)	-	-	-	-	-	-
$[Ac(DOTA)(H_2O)]^{-}$	M06-HF/ 6-311G(d)	-1885.515	-1885.053	-1885.109	183.086	-1885.643	-1885.693
	B3LYP/ 6-311G(d)	-	-	-	-	-	-
[Ac(3p-*C*-DEPA)]$^{2-}$	M06-HF/ 6-311G(d)	-2723.788	-2723.070	-2723.147	254.254	-2724.068	-2724.104
	B3LYP/ 6-311G(d)	-	-	-	-	-	-
$[Bi(H_2O)_9]^{3+}$	M06-HF/ 6-311G(d)	-691.871	-691.640	-691.694	168.023	-692.412	-692.486
	B3LYP/ 6-311G(d)	-692.094	-691.869	-691.926	178.930	-692.685	-692.705
$[Bi(DOTA)(H_2O)]^{-}$	M06-HF/ 6-311G(d)	-1526.597	-1526.134	-1526.192	182.735	-1526.726	-1526.732
	B3LYP/ 6-311G(d)	-1526.929	-1526.480	-1526.539	189.387	-1527.015	-1527.051
[Bi(3p-*C*-DEPA)]$^{2-}$	M06-HF/ 6-311G(d)	-2364.860	-2364.144	-2364.221	254.532	-2365.136	-2365.135
	B3LYP/ 6-311G(d)	-2365.409	-2364.711	-2364.788	260.718	-2365.624	-2365.658
$[Lu(H_2O)_9]^{3+}$	M06-HF/ 6-311G(d)	-691.871	-691.640	-691.694	178.930	-725.209	-725.822
	B3LYP/ 6-311G(d)	-725.209	-725.328	-725.378	158.875	-726.126	-726.189
$[Lu(DOTA)(H_2O)]^{-}$	M06-HF/ 6-311G(d)	-1559.923	-1559.459	-1559.514	176.523	-1560.060	-1560.068
	B3LYP/ 6-311G(d)	-1560.377	-1559.926	-1559.983	183.437	-1560.482	-1560.512
[Lu(3p-*C*-DEPA)]$^{2-}$	M06-HF/ 6-311G(d)	-2398.161	-2397.442	-2397.518	251.566	-2398.447	-2398.450
	B3LYP/ 6-311G(d)	-2398.831	-2398.133	-2398.212	262.653	-2399.090	-2399.096

[a]6-311G(d)basis set was used for all atoms except Ac, Bi, and Lu.

`**Table S2.** Compiled $\Delta G°_g$, ΔG_{aq}, and log K_1 calculated in this work

Equilibrium	Functional/ Basis Sets[a]	$\Delta G°_g$ (kcal/mol)	ΔG_{aq} (COSMO) (kcal/mol)	ΔG_{aq} (SMD) (kcal/mol)	log K_1	log K_1 (SMD)	log K_1 (COSMO)
$[Ac(H_2O)_9]^{3+}$ + $[DOTA]^{4-}$ ⇌ $[Ac(DOTA)(H_2O)]^{-}$	M06-HF/ 6-311G(d)	-1126.46	-38.44	-31.04	815.83	22.75	28.17
	B3LYP/ 6-311G(d)	-	-	-	-	-	-
$[Ac(H_2O)_9]^{3+}$ + [3p-*C*-DEPA]$^{5-}$ ⇌ [Ac(3p-*C*-DEPA]$^{2-}$	M06-HF/ 6-311G(d)	-882.54	-93.56	-82.91	637.07	60.76	68.57
	B3LYP/ 6-311G(d)	-	-	-	-	-	-
$[Bi(H_2O)_9]^{3+}$ + $[DOTA]^{4-}$ ⇌ $[Bi(DOTA)(H_2O)]^{-}$	M06-HF/ 6-311G(d)	-1150.60	-74.86	-64.06	833.52	46.95	54.86
	B3LYP/ 6-311G(d)	-847.57	-94.88	-71.71	611.44	52.55	69.54
$[Bi(H_2O)_9]^{3+}$ + [3p-*C*-DEPA]$^{5-}$ ⇌ [Bi(3p-*C*-DEPA]$^{2-}$	M06-HF/ 6-311G(d)	-901.42	-65.03	-80.37	650.910	40.51	47.66
	B3LYP/	-1258.34	-80.24	-61.42	912.486	45.01	58.81

	6-311G(d)						
$[Lu(H_2O)_9]^{3+}$ + $[DOTA]^{4-}$ ⇌ $[Lu(DOTA)(H_2O)]^-$	M06-HF/ 6-311G(d)	-876.24	-81.97	-62.23	830.72	41.38	46.13
	B3LYP/ 6-311G(d)	-888.91	-92.76	-74.44	607.82	45.44	56.04
$[Lu(H_2O)_9]^{3+}$ + [3p-*C*-DEPA]$^{5-}$ ⇌ [Lu(3p-*C*-DEPA)]$^{2-}$	M06-HF/ 6-311G(d)	-882.03	-65.03	-39.96	636.69	20.93	29.44
	B3LYP/ 6-311G(d)	-785.70	-54.41	-43.91	485.44	24.72	29.79

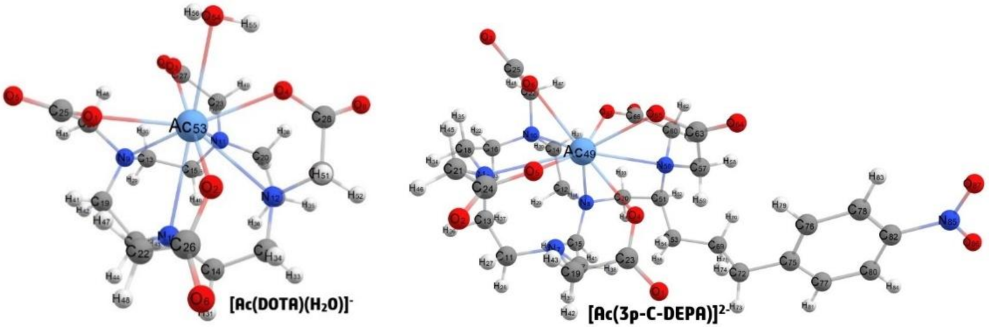

Figure S1. DFT-optimized structure and cartesian coordinate of $[Ac(DOTA)(H_2O)]^-$, and [Ac(3p-*C*-DEPA]$^{2-}$ complex.

Cartesian coordinates of the most important optimized structures (Angstroms).

Cartesian coordinate of $[Ac(DOTA)(H_2O)]^-$ complex

Atom	X	Y	Z
O	-1.68064	1.51603	-1.19104
O	1.36684	1.67614	-1.3471
O	-1.7094	-1.49417	-1.00593
O	1.20431	-1.78731	-1.35848
O	-3.86496	1.90424	-1.45026
O	2.28492	3.69355	-1.06288
O	-3.05131	-3.06954	-0.1773
O	3.05819	-3.03208	-1.37481
N	-1.99345	0.41115	1.18691
N	0.56713	1.89211	1.28443
N	-0.05136	-1.87944	1.21162
N	2.3573	-0.26277	0.60049
C	-2.08176	-0.71396	2.13213
C	1.9205	1.82971	1.86998
C	-0.73411	-1.39349	2.41892
C	2.91805	1.02609	1.02381
C	-0.43482	2.00299	2.35112
C	2.49634	-1.18577	1.743
C	-1.84284	1.71917	1.84357
C	1.30768	-2.34006	1.55279
C	-3.20137	0.50354	0.34294
C	0.48532	3.04672	0.35938
C	-0.79088	-2.98972	0.57647
C	3.16943	-0.81601	-0.49893
C	-2.92338	1.39931	-0.88338
C	1.47864	2.82472	-0.80282
C	-1.9821	-2.49802	-0.26773
C	2.44149	-2.0097	-1.1504
H	-2.5041	-0.38297	3.0881
H	-2.76054	-1.45701	1.71985
H	2.32083	2.8399	2.00108
H	1.84292	1.38181	2.85893
H	3.82331	0.87008	1.62523
H	3.19694	1.5874	0.1397
H	3.28753	-1.61517	1.93099
H	2.0162	-0.41739	2.62698
H	1.28356	-2.92581	2.48028

H	1.66143	-2.99231	0.7625
H	-0.92043	-2.23183	3.10266
H	-0.06744	-0.69692	2.9202
H	-2.11502	2.48245	1.11931
H	-2.54346	1.80003	2.68359
H	-0.18633	1.29557	3.13922
H	-0.41796	3.0068	2.79379
H	-4.0429	0.9287	0.89614
H	-3.46926	-0.4767	-0.03987
H	-0.5085	3.08837	-0.08052
H	0.73502	3.9757	0.87638
H	-0.11603	-3.46238	-0.13408
H	-1.14315	-3.71003	1.31834
H	3.2566	-0.03974	-1.25782
H	4.15471	-1.13385	-0.15058
Ac	0.00263	0.00654	-0.37675
O	-0.3407	-0.28744	-2.95167
H	0.41409	-0.36032	-3.54038
H	-1.23183	-0.33131	-3.30603

Cartesian coordinate of $[Ac(3p\text{-}C\text{-}DEPA)]^{2-}$complex

Atom	X	Y	Z
O	1.91169	2.96331	-0.7639
O	-3.52888	4.13434	-1.88834
O	-6.04251	-1.93069	-1.24094
O	0.4563	1.31509	-0.99115
O	-2.68296	2.08946	-1.81913
O	-4.175	-0.92992	-1.41062
N	-1.08923	2.47916	0.94305
N	-0.95274	-0.44633	2.27234
N	-4.09991	1.69751	0.6138
N	-3.88652	-1.24455	1.38838
C	-2.25039	3.22373	1.39256
C	-2.03857	-1.06193	3.08253
C	-3.51367	2.44992	1.71284
C	-3.07887	-1.93975	2.38449
C	-0.61252	0.85559	2.88697
C	-4.80179	-0.2878	1.99571
C	-0.19672	2.03869	2.01116
C	-5.20444	0.87286	1.06274
C	-0.29956	3.29293	0.00571
C	0.17142	-1.42232	2.25569
C	-4.56077	2.5641	-0.47186
C	-4.62442	-2.19071	0.54239
C	0.80621	2.46753	-0.65539
C	-3.48684	2.99116	-1.48647
C	-5.04301	-1.63325	-0.82985
H	-2.00278	3.82391	2.28792
H	-2.48882	3.97075	0.63148
H	-1.59808	-1.6624	3.88116
H	-2.5648	-0.27042	3.58876
H	-3.71062	-2.33859	3.19006
H	-2.61386	-2.78514	1.91316
H	-5.72394	-0.78886	2.30417
H	-4.3716	0.09849	2.90719
H	-5.92938	1.48314	1.64811
H	-5.72906	0.48512	0.23596
H	-4.24255	3.17068	2.10724
H	-3.30374	1.76628	2.51956
H	0.75628	1.83079	1.56825
H	-0.01683	2.86322	2.7127
H	-1.46069	1.18256	3.46702
H	0.19068	0.72266	3.61547
H	0.14737	4.15028	0.51074
H	-0.94852	3.65598	-0.77721
H	0.66594	-1.38424	3.23139
H	-5.28721	2.0106	-1.05301
H	-5.05739	3.455	-0.08587
H	-3.97932	-3.02838	0.33342
H	-5.51456	-2.55613	1.05401
Ac	-1.78158	-0.15115	-0.87891
H	-0.28431	-2.39729	2.20492
C	1.32082	-1.43388	1.20457

H	1.91767	-2.27551	1.57409
C	2.24413	-0.20869	1.28618
H	1.91366	0.54432	0.59523
H	2.16171	0.21317	2.28418
N	0.8441	-1.77421	-0.14939
C	1.69965	-1.4282	-1.28788
H	2.55988	-2.09113	-1.36499
H	2.04552	-0.41317	-1.20481
C	0.50303	-3.19744	-0.23628
H	1.20046	-3.80908	0.33346
H	0.5824	-3.51019	-1.26913
C	0.95994	-1.47726	-2.64321
O	1.62849	-1.76668	-3.60744
O	-0.26488	-1.18097	-2.61969
C	-0.92581	-3.53207	0.17714
O	-1.77893	-2.69632	-0.19352
O	-1.12471	-4.54961	0.81421
C	3.43104	-0.48648	1.04185
H	3.87593	-1.04256	0.12579
H	4.13489	-1.10578	1.84231
C	4.51593	0.83367	0.95706
H	4.43127	1.36763	1.89988
H	4.06127	1.46724	0.20473
C	5.97513	0.62188	0.63608
C	6.40513	0.55085	-0.68783
C	6.92093	0.46941	1.64749
C	7.7331	0.33566	-0.99649
H	5.69034	0.66872	-1.48159
C	8.25323	0.25453	1.35933
H	6.60915	0.52419	2.57531
C	8.64487	0.19121	0.03478
H	8.06094	0.28325	-2.01483
H	8.979	0.14074	2.13878
N	10.05122	-0.02657	-0.27835
O	10.81281	-0.1518	0.62912
O	10.37014	-0.06704	-1.4227

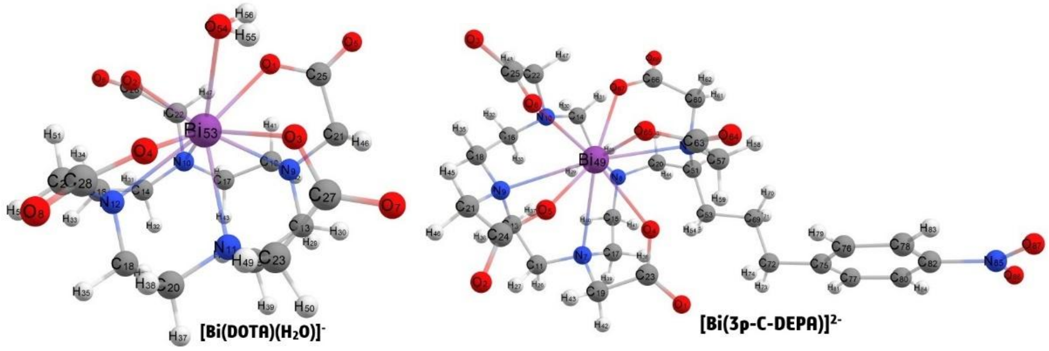

Figure S2. DFT-optimized structure and cartesian coordinate of $[Bi(DOTA)(H_2O)]^-$, and $[Bi(3p\text{-}C\text{-}DEPA]^{2-}$ complex.

Cartesian coordinate of $[Bi(DOTA)(H_2O)]^-$ complex

Atom	X	Y	Z
O	-1.95557	1.48216	-1.37571
O	1.57145	1.80982	-1.42933
O	-1.84193	-1.83925	-0.73787
O	1.35152	-1.97195	-1.251
O	-4.14113	1.96405	-1.36698
O	2.53059	3.77595	-0.96917
O	-3.16521	-3.15769	0.48567
O	3.42563	-3.09277	-0.77942
N	-2.0572	0.59615	1.1513
N	0.55363	2.07843	1.20971
N	-0.08172	-1.76752	1.24825
N	2.40216	-0.18642	0.76689
C	-2.07277	-0.4052	2.23105
C	1.86331	1.99753	1.88173

C	-0.69796	-1.01892	2.55268
C	2.90685	1.14248	1.1446
C	-0.51445	2.29791	2.1959
C	2.29048	-1.05071	1.95228
C	-1.90101	1.97334	1.64673
C	1.27964	-2.19648	1.81646
C	-3.30529	0.56202	0.35988
C	0.5516	3.17471	0.21354
C	-0.87112	-2.96218	1.10184
C	3.31754	-0.77379	-0.22914
C	-3.15471	1.42763	-0.91826
C	1.66386	2.93792	-0.83503
C	-2.09771	-2.6423	0.22148
C	2.74867	-2.08763	-0.80451
H	-2.4552	0.04243	3.1567
H	-2.76088	-1.20167	1.95726
H	2.28631	2.99978	2.01119
H	1.40441	1.58395	2.87645
H	3.77672	1.0419	1.80831
H	3.23661	1.64981	0.24514
H	3.26463	-1.50278	2.1744
H	2.02478	-0.43332	2.80682
H	1.25553	-2.7293	2.77724
H	1.62545	-2.89398	1.06121
H	-0.82638	-1.68208	3.21971
H	-0.00952	-0.22868	2.83435
H	-2.12467	2.64649	0.82319
H	-2.63665	2.16707	2.43808
H	-0.32274	1.68209	3.07151
H	-0.51585	3.34225	2.53305
H	-4.15297	0.94369	0.93457
H	-3.5011	-0.45861	0.03782
H	-0.40386	3.17972	-0.31183
H	0.72825	4.13649	0.7009
H	-0.23901	-3.60904	0.49588
H	-1.20006	-3.49695	1.9968
H	3.40401	-0.0635	-1.05108
H	4.29951	-0.97466	0.20628
Bi	-0.04468	-0.00608	-0.78182
O	-0.39929	-1.57068	-2.98631
H	0.35606	-2.12535	-2.73026
H	-1.20499	-1.97397	-2.63167

Cartesian coordinate of $[Bi(3p\text{-}C\text{-DEPA})]^{2-}$ complex

**Atom**	**X**	**Y**	**Z**
O	1.79868	3.24656	0.30833
O	-3.08623	4.03309	-1.9986
O	-5.20953	-2.22282	-2.4701
O	0.64525	1.44678	-0.31077
O	-2.04833	2.05968	-1.89276
O	-3.37625	-1.02394	-2.03287
N	-1.47895	2.38458	1.04895
N	-1.40406	-0.49184	2.32263
N	-4.09683	1.25769	-0.10979
N	-3.76033	-1.59093	0.66997
C	-2.80727	2.98984	1.19617
C	-2.59166	-1.2446	2.81978
C	-3.97247	2.00002	1.14371
C	-3.24593	-2.21717	1.86013
C	-1.36074	0.79827	3.05378
C	-4.98446	-0.80793	0.98594
C	-0.83972	2.04037	2.32269
C	-5.22656	0.32917	-0.00734
C	-0.58519	3.33913	0.35995
C	-0.22514	-1.36153	2.59945
C	-4.32461	2.1362	-1.26381
C	-4.12273	-2.56641	-0.38114
C	0.75433	2.64445	0.07661
C	-3.0407	2.82924	-1.7696
C	-4.26708	-1.88949	-1.76603
H	-2.87407	3.53128	2.15103
H	-2.93926	3.72321	0.4036

H	-2.30032	-1.77967	3.73935
H	-3.34954	-0.52034	3.10159
H	-4.04843	-2.74175	2.4317
H	-2.54163	-3.00959	1.54383
H	-5.87309	-1.43358	0.97472
H	-4.91041	-0.40419	1.99266
H	-6.13251	0.87121	0.30864
H	-5.41827	-0.08729	-0.99018
H	-4.89608	2.57155	1.33407
H	-3.85636	1.27962	1.94836
H	0.21982	1.92998	2.14001
H	-0.94837	2.87705	3.03136
H	-2.36807	1.03384	3.38754
H	-0.75663	0.68758	3.96364
H	-0.39739	4.21981	0.98291
H	-1.02902	3.62756	-0.58984
H	-0.01776	-1.31768	3.67937
H	-4.65388	1.50345	-2.08642
H	-5.08079	2.89886	-1.04628
H	-3.30028	-3.26983	-0.45942
H	-5.05935	-3.04609	-0.1555
Bi	-1.42306	-0.10858	-0.68997
H	-0.54174	-2.38011	2.38647
C	1.14856	-1.18014	1.89434
H	1.73477	-1.99164	2.35541
C	1.88868	0.12504	2.2389
H	1.57667	0.88146	1.52809
H	1.60203	0.44407	3.24286
N	1.06308	-1.42822	0.44155
C	2.1467	-0.89378	-0.38607
H	3.04337	-1.52112	-0.34632
H	2.37729	0.12269	-0.09839
C	0.9598	-2.86493	0.15874
H	1.65242	-3.44796	0.77357
H	1.18943	-3.01343	-0.89467
C	1.75542	-0.75984	-1.88051
O	2.66765	-0.59105	-2.64375
O	0.52114	-0.82054	-2.15396
C	-0.46772	-3.37914	0.33984
O	-1.33308	-2.63699	-0.20383
O	-0.68234	-4.40471	0.98147
C	3.42966	-0.01939	2.13938
H	3.67928	-0.89085	1.55962
H	3.84886	-0.19519	3.15187
C	4.10459	1.23054	1.52478
H	4.32185	1.96724	2.29801
H	3.43167	1.71343	0.81539
C	5.38229	0.83347	0.82072
C	5.35069	0.43729	-0.52256
C	6.59783	0.80183	1.50957
C	6.50846	0.01357	-1.15938
H	4.4238	0.44268	-1.08424
C	7.76604	0.38814	0.88938
H	6.62477	1.11039	2.54552
C	7.69357	0.00127	-0.43897
H	6.47106	-0.30058	-2.1906
H	8.71296	0.36124	1.4035
N	8.92789	-0.43442	-1.10097
O	9.94544	-0.4525	-0.4481
O	8.87033	-0.75138	-2.26034

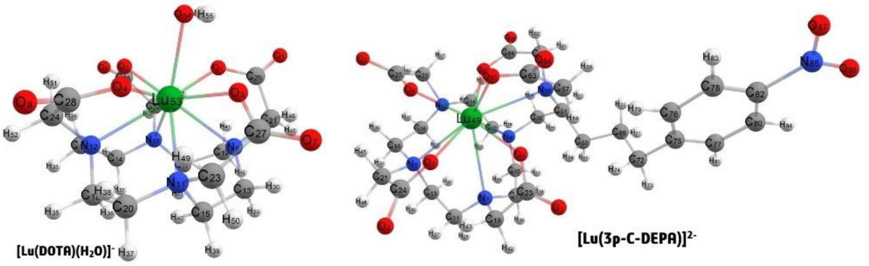

Figure S3. DFT-optimized structure and cartesian coordinate of $[Lu(DOTA)(H_2O)]^-$, and $[Lu(3p\text{-}C\text{-}DEPA)(H_2O)]^{2-}$ complex.

Cartesian coordinate of $[Lu(DOTA)(H_2O)]^-$ complex

Atom	X	Y	Z
O	-2.05878	1.23606	-1.27348
O	1.34118	2.40801	-0.96505
O	-1.45468	-1.88311	-0.87494
O	2.13565	-1.4288	-1.31244
O	-4.24121	1.8056	-1.18798
O	2.05406	4.50508	-0.49721
O	-2.61107	-3.72968	-0.2768
O	4.12719	-2.49963	-1.20008
N	-1.97221	0.23238	1.17576
N	0.43545	1.96319	1.50633
N	0.10791	-1.90235	1.32226
N	2.44073	-0.11092	0.9889
C	-2.0622	-0.90293	2.15145
C	1.69625	1.86671	2.32103
C	-0.70549	-1.52672	2.53113
C	2.87222	1.19414	1.61241
C	-0.66781	1.93211	2.52703
C	2.35382	-1.07711	2.13865
C	-2.02494	1.51517	1.9571
C	1.46392	-2.29137	1.83634
C	-3.31616	0.23367	0.45457
C	0.45137	3.39315	0.98427
C	-0.4947	-3.20639	0.83018
C	3.69963	-0.55641	0.21939
C	-3.23728	1.18609	-0.76352
C	1.37719	3.4971	-0.2592
C	-1.64016	-2.96464	-0.18416
C	3.33422	-1.61359	-0.85501
H	-2.56554	-0.59325	3.10865
H	-2.68147	-1.72803	1.68817
H	2.05987	2.88553	2.65067
H	1.49661	1.3018	3.28545
H	3.70676	1.05891	2.35347
H	3.23518	1.886	0.79578
H	3.36126	-1.4781	2.43581
H	1.93573	-0.54801	3.03875
H	1.38818	-2.91053	2.77123
H	1.97976	-2.90619	1.04038
H	-0.91755	-2.41963	3.18337
H	-0.1129	-0.79837	3.15038
H	-2.39093	2.32682	1.26091
H	-2.75193	1.45427	2.81431
H	-0.41419	1.20231	3.34788
H	-0.79737	2.93988	3.01352
H	-4.15136	0.50924	1.14595
H	-3.49752	-0.79817	0.03723
H	-0.58801	3.66018	0.64996
H	0.76419	4.12864	1.76966
H	0.28966	-3.78134	0.26386
H	-0.85527	-3.84329	1.67863
H	4.11269	0.32551	-0.34399
H	4.50549	-0.94422	0.89403
Lu	0.18332	0.11636	-0.57983
O	-0.80491	-0.13632	-2.93235
H	-1.46856	-0.78859	-2.67364
H	-1.28929	0.69278	-3.00676

Figure S4. DFT-optimized structure and cartesian coordinate of $[Lu(3p\text{-}C\text{-}DEPA)]^{2-}$complex.

Cartesian coordinate of $[Lu(3p\text{-}C\text{-}DEPA)]^{2-}$complex

Atom	X	Y	Z
O	-2.05878	1.23606	-1.27348
O	1.34118	2.40801	-0.96505
O	-1.45468	-1.88311	-0.87494
O	2.13565	-1.4288	-1.31244
O	-4.24121	1.8056	-1.18798

O	2.05406	4.50508	-0.49721
O	-2.61107	-3.72968	-0.2768
O	4.12719	-2.49963	-1.20008
N	-1.97221	0.23238	1.17576
N	0.43545	1.96319	1.50633
N	0.10791	-1.90235	1.32226
N	2.47073	-0.11092	0.9889
C	-2.0622	-0.90293	2.15145
C	1.69625	1.86671	2.33103
C	-0.70549	-1.52672	2.53113
C	2.83222	1.19414	1.61241
C	-0.66781	1.93211	2.52703
C	2.35382	-1.07711	2.13865
C	-2.02494	1.51517	1.9171
C	1.46392	-2.29137	1.83634
C	-3.31616	0.23367	0.45457
C	0.45137	3.39315	0.98427
C	-0.4947	-3.20639	0.83018
C	3.69963	-0.55641	0.21939
C	-3.25728	1.18609	-0.76352
C	1.37719	3.4971	-0.2592
C	-1.64016	-2.96464	-0.18416
C	3.33422	-1.61359	-0.85501
H	-2.56554	-0.59325	3.10865
H	-2.68147	-1.72803	1.68817
H	2.05987	2.88553	2.65067
H	1.49661	1.3018	3.28545
H	3.70676	1.05891	2.35347
H	3.23518	1.886	0.79578
H	3.36126	-1.4781	2.44581
H	1.93573	-0.54801	3.03875
H	1.38818	-2.91053	2.77123
H	1.97976	-2.90619	1.04038
H	-0.91755	-2.41963	3.18337
H	-0.1129	-0.79837	3.15038
H	-2.39093	2.32682	1.26091
H	-2.55193	1.45427	2.81431
H	-0.41419	1.20231	3.34788
H	-0.79737	2.93988	3.01352
H	-4.15136	0.50924	1.14595
H	-3.49752	-0.79817	0.03723
H	-0.58801	3.67018	0.64996
H	0.76419	4.12864	1.76966
H	0.28966	-3.78134	0.26386
H	-0.85527	-3.84329	1.64863
H	4.11269	0.32551	-0.34399
H	4.50549	-0.94422	0.89403
Lu	0.18332	0.11636	-0.57983
O	-0.80491	-0.13632	-2.93235
H	-1.46856	-0.78859	-2.67364
H	-1.28929	0.69278	-3.00676

Table S3. Atomic charge distribution of donor atoms in $[DOTA]^{4-}$ and $[3p\text{-}C\text{-}DEPA]^{5-}$

**Chelators**	**Donor Atom**	**Natural Population Analysis (NPA)**	**Chelators**	**Donor Atom**	**Natural Population Analysis (NPA)**
$[DOTA]^{4-}$	O1	-0.88640	[3p-*C*-DEPA]$^{5-}$	O1	-0.88879
	O2	-0.88863		O2	-0.88015
	O3	-0.88892		O3	-0.88636
	O4	-0.85891		O4	-0.86616
	O5	-0.85908		O5	-0.87407
	O6	-0.86149		O6	-0.86812
	O7	-0.85854		O50	-0.89235
	O8	-0.88575		O51	-0.86811
	Average O	-0.87347		O53	-0.86789
	N9	-0.59168		O54	-0.88691
	N10	-0.58181		Average O	-0.87789
	N11	-0.59308		N7	-0.60188
	N12	-0.60405		N8	-0.59975
	Average N	-0.59266		N9	-0.60094

				N10	-0.59290
				Average N cyclen	-0.59887
				N45	-0.59119

Table S4. Charge distribution of the ion center in the [DOTA]$^{4-}$ and [3p-C-DEPA]$^{5-}$ complexes

**Chelators**	**Ions**	**Natural Population Analysis (NPA)**	**Chelators**	**Ions**	**Natural Population Analysis (NPA)**
[DOTA]$^{4-}$	Lu^{3+}	1.60296	[3p-*C*-DEPA]$^{5-}$	Lu^{3+}	1.77991
	Bi^{3+}	2.25461		Bi^{3+}	2.27544
	Ac^{3+}	1.42212		Ac^{3+}	1.36652

Table S5. Bond order between the ion and donor atoms in the [DOTA]$^{4-}$ complex.

**Chelators**	**Ions**	**Bond**	**Wiberg Bond Index (WBI)**
[DOTA]$^{4-}$	Lu^{3+}	O1-Lu	0.2712
		O2-Lu	0.2852
		O3-Lu	0.2705
		O4-Lu	0.2836
		N9-Lu	0.1383
		N10-Lu	0.1453
		N11-Lu	0.1422
		N12-Lu	0.1368
		O54-Lu	0.2343
	Bi^{3+}	O1-Bi	0.1512
		O2-Bi	0.1539
		O3-Bi	0.1860
		O4-Bi	0.1971
		N9-Bi	0.0962
		N10-Bi	0.0814
		N11-Bi	0.1133
		N12-Bi	0.0921
		O54-Bi	0.1448
	Ac^{3+}	O1-Ac	0.3428
		O2-Ac	0.3341
		O3-Ac	0.3211
		O4-Ac	0.3254
		N9-Ac	0.1276
		N10-Ac	0.1313
		N11-Ac	0.1270
		N12-Ac	0.1283
		O54-Ac	0.2767

Table S6. Bond order between the ion and donor atoms in the $[3p\text{-}C\text{-}DEPA]^{5-}$ complex

Chelators	Ions	Bond	Wiberg Bond Index (WBI)
$[3p\text{-}C\text{-}DEPA]^{5-}$	Lu^{3+}	O4-Lu	0.1834
		O5-Lu	0.2176
		O6-Lu	0.2259
		O65-Lu	0.2380
		O67-Lu	0.1878
		N7-Lu	0.0967
		N8-Lu	0.1289
		N9-Lu	0.0931
		N10-Lu	0.0857
		N56-Lu	0.0823
	Bi^{3+}	O4-Bi	0.1344
		O5-Bi	0.1919
		O6-Bi	0.2060
		O65-Bi	0.2274
		O67-Bi	0.1510
		N7-Bi	0.0542
		N8-Bi	0.0615
		N9-Bi	0.0629
		N10-Bi	0.0527
		N56-Bi	0.0715
	Ac^{3+}	O4-Ac	0.2827
		O5-Ac	0.3160
		O6-Ac	0.3251
		O65-Ac	0.3248
		O67-Ac	0.2826
		N7-Ac	0.1223
		N8-Ac	0.1364
		N9-Ac	0.1200
		N10-Ac	0.1207
		N56-Ac	0.1125
